# The PR/SET Domain Zinc Finger Protein Prdm4 Regulates Gene Expression in Embryonic Stem Cells but Plays a Nonessential Role in the Developing Mouse Embryo

**DOI:** 10.1128/MCB.00498-13

**Published:** 2013-10

**Authors:** Debora Bogani, Marc A. J. Morgan, Andrew C. Nelson, Ita Costello, Joanna F. McGouran, Benedikt M. Kessler, Elizabeth J. Robertson, Elizabeth K. Bikoff

**Affiliations:** Sir William Dunn School of Pathology, University of Oxford, Oxford, United Kingdoma; Henry Wellcome Building for Molecular Physiology, Nuffield Department of Medicine, University of Oxford, Oxford, United Kingdomb; Target Discovery Institute, Nuffield Department of Medicine, University of Oxford, Oxford, United Kingdomc

## Abstract

Prdm4 is a highly conserved member of the Prdm family of PR/SET domain zinc finger proteins. Many well-studied Prdm family members play critical roles in development and display striking loss-of-function phenotypes. Prdm4 functional contributions have yet to be characterized. Here, we describe its widespread expression in the early embryo and adult tissues. We demonstrate that DNA binding is exclusively mediated by the Prdm4 zinc finger domain, and we characterize its tripartite consensus sequence via SELEX (systematic evolution of ligands by exponential enrichment) and ChIP-seq (chromatin immunoprecipitation-sequencing) experiments. In embryonic stem cells (ESCs), *Prdm4* regulates key pluripotency and differentiation pathways. Two independent strategies, namely, targeted deletion of the zinc finger domain and generation of a EUCOMM LacZ reporter allele, resulted in functional null alleles. However, homozygous mutant embryos develop normally and adults are healthy and fertile. Collectively, these results strongly suggest that Prdm4 functions redundantly with other transcriptional partners to cooperatively regulate gene expression in the embryo and adult animal.

## INTRODUCTION

PRDM family members share a characteristic modular structure with the N-terminal PR/SET domain and a variable number of C-terminal zinc finger (ZF) repeats (1–3). Gene targeting experiments have revealed critical roles as cell-type-specific transcriptional regulators of mouse development. For example, targeted loss of *Prdm1* disrupts germ cell specification, forelimb patterning, placental morphogenesis, and postnatal reprogramming of intestinal enterocytes (4–9). Similarly, *Prdm14* plays an essential role in specification of the germ cell lineage (10). Prdm16 promotes brown fat identity and represses the alternative skeletal muscle cell fate (11). Interestingly, a *Prdm16* splice site mutation that creates a hypomorphic allele causes craniofacial skeletal defects (12). Prdm3 and Prdm16 regulate maintenance of hematopoietic stem cell function (13, 14). Prdm9 governs meiotic recombination (15–17). Deregulated expression of several Prdm family members, including PRDM1, PRDM2/RIZ1, PRDM3, PRDM5, PRDM14, and PRDM16, has been implicated in human cancers (1, 3).

The evolutionarily conserved PR/SET domain is 20 to 30% identical to the SET domain found in numerous histone lysine methyltransferases (HMTs) that modify nucleosome structure (18, 19). Several Prdms, including Prdm3, Prdm9, and Prdm16, act directly as histone H3 methyltransferases (1, 3, 20), while others seem to lack intrinsic catalytic activity. Rather, their ability to regulate target gene expression is probably mediated via recruitment of epigenetic partners such as G9a, histone deacetylases (HDACs), Lsd1, and the arginine methyltransferase Prmt5 (21).

The zinc finger (ZF) repeats typically function to mediate nuclear import and sequence-specific DNA binding (1, 3, 21). Unique consensus binding motifs recognized by several family members have been previously characterized. For example, Prdm9 recognizes a 13-mer motif enriched at recombination hot spots (22). Consistent with its proposed role in osteogenic differentiation (23), Prdm5 predominantly binds within the exonic regions of collagen genes in association with RNA polymerase II. Prdm14 binding sites in embryonic stem cells (ESCs) substantially overlap those occupied by Nanog and Oct4, in keeping with its ability to protect ESCs from entering extraembryonic endoderm fates and ensure pluripotency (24). Blimp1/Prdm1 directly silences the promoter regions of key transcription factors such as c-myc, Pax5, and CIITA expressed in B lymphocytes to dramatically shift the developmental program toward a terminal plasma cell fate (25). Similarly, Prdm1 directly represses c-myc expression in macrophages and sebaceous gland progenitors to arrest cell cycle progression, but c-myc is not a target in T cells (26–28). Rather, Prdm1 silences expression of an essential cytokine, interleukin-2 (IL-2), necessary for T cell proliferation (29). A distinct set of Blimp1/Prdm1 transcriptional targets has been characterized in the skin epidermis (30). Genome-wide ChIP-chip (chromatin immunoprecipitation with microarray technology) experiments identified an extended set of Blimp1/Prdm1-occupied promoters in human myelomas (31). Collectively, these experiments suggest that a dynamic process governs cell-type-specific Blimp1/Prdm1 target site selection within the nucleus.

Considerably less is known about transcriptional targets and the functional roles played by other Prdm family members. In particular, Prdm4 was originally identified in a yeast two-hybrid screen as a factor that interacts with the p75 neurotrophin receptor and displays a dynamic pattern of expression in the developing nervous system (32–34). A recent report suggests that Prdm4 controls proliferation and differentiation in neural stem cells (33). Prdm4 has a long N-terminal domain including a zinc knuckle, followed by the PR/SET domain and six C2H2 ZFs (32, 35). As for other PRDMs, Prdm4 interacts with HDACs (36), as well as Prmt5 (33), and its zinc fingers mediate nuclear import (36). However, developmentally regulated expression of *Prdm4* outside the central nervous system and characterization of a loss-of-function allele have yet to be reported.

Here, we describe widespread *Prdm4* expression during early mouse development and throughout adult tissues. The strongest expression was detectable in reproductive tissues. To characterize the Prdm4 DNA binding consensus motif, we performed systematic evolution of ligands by exponential enrichment (SELEX) experiments. To assess Prdm4 occupancy in mouse embryonic stem cells (ESCs), we performed a genome-wide screen via chromatin immunoprecipitation followed by high-throughput sequencing (ChIP-seq). Interestingly, Prdm4 displays a marked bias toward binding proximally to transcription start sites (TSSs). As expected, targeted deletion of the zinc finger domain (ZFD) encoded by exons 9 to 11 disrupts nuclear import and DNA binding. Expression microarray experiments reveal significant changes in Prdm4-dependent transcriptional profiles in ESCs consistent with a functional role in governing pluripotency and differentiation. Unexpectedly, however, homozygous mutant embryos develop normally and adults are healthy and fertile. We conclude that Prdm4 influences the core regulatory circuitry in cultured ESCs but plays a nonessential role *in vivo*.

## MATERIALS AND METHODS

### ISH and histology.

Embryonic day 6.5 (E6.5) to E9.5 embryos were fixed with 4% paraformaldehyde (PFA) overnight at 4°C, and whole-mount *in situ* hybridization (ISH) analysis was carried out according to standard protocols (37). Prdm4 riboprobes spanning nucleotides (nt) 3081 to 3506 of the sequence with accession no. NM_181650 (kindly provided by Adrian Moore, RIKEN Brain Science Institute, Wako, Saitama, Japan) and the full-length cDNA (IMAGE clone 8862431) were used. For histology, embryos were postfixed in 4% PFA, dehydrated through an ethanol series, embedded in paraffin, sectioned at 8 μm, and eosin counterstained. For section ISH, tissue was fixed overnight in 4% paraformaldehyde, dehydrated through an ethanol series, embedded in paraffin, sectioned at 7 to 8 μm, and processed according to standard protocols. Testes and ovaries were fixed overnight in 4% PFA, dehydrated in ethanol, embedded in paraffin, sectioned at 8 μm, and stained with hematoxylin and eosin.

### Northern blot analysis.

Total RNA was extracted from individual organs of 4-week-old C57BL/6J mice using the TRIzol method (Gibco/BRL). Total RNA (10 μg/lane) was size fractionated on a 1% agarose-formaldehyde gel, transferred onto Hybond N membranes (GE Healthcare), and probed with a ^32^P-random-primed full-length cDNA Prdm4 fragment.

### Gene targeting.

The *Prdm4^ΔZF^* targeting vector was generated by ligating a 6.9-kb 5′ homology region (EcoRI-XhoI) and a 4.6-kb 3′ homology region (AfeI-EcoRV) from the bMQ362d07 bacterial artificial chromosome (BAC) (Source Bioscience, Cambridge, United Kingdom) and the *loxP*-flanked *pgk-hygromycin* cassette (2.1 kb) (38) into a modified version of pBSII-KS(−) (Stratagene). The *hsv-thymidine kinase* (*hsv-tk*) cassette was added outside the 3′ homology region. A NotI-linearized targeting vector (15 μg) was introduced into CCE ESCs by electroporation. Drug-resistant colonies selected in the presence of hygromycin (1.5 μg/ml) and 1-[2′-deoxy-2′-fluoro-β-d-arabinofuranosyl]-5-iodouracil (FIAU) (0.1 μg/ml) were screened by Southern blotting using the restriction enzyme and probe combinations shown in Fig. 5. For excision of the loxP-flanked *pgk-hygromycin* cassette, correctly targeted *Prdm4^+/ΔZF^* clones were transiently transfected with p*MC1Cre* and subsequently screened by Southern blotting. Three excised clones were retargeted to generate doubly targeted *Prdm4^ΔZF/ΔZF^* clones. For biochemical studies, wild-type and *Prdm4^ΔZF/ΔZF^* clones were adapted to grow under feeder-free conditions on gelatin-coated plates in medium containing 1,000 U/ml of leukemia inhibitory factor (LIF). To generate animals carrying the targeted allele, C57BL/6J blastocysts were injected with 12 to 14 *Prdm4^+/ΔZF^* ESCs and transferred into E2.5 pseudopregnant foster females. The following primers and cycling conditions were used for PCR genotyping to distinguish the wild-type and Δ*ZF* alleles: common primer, TGC TTA CAG AGG GTA TGG TAT GA; wild-type primer, GGC CAC CAA ATT CTG TTC TTC A; mutant primer, GAT GGT CAG GTA CAC CCA AGA; 60°C annealing temperature, 40 cycles. The *Prdm4^TA^* (Prdm4EUCOMM) targeting vector (project ID 45696) was obtained from Helmholtz Zentrum München Deutsches Forschungszentrum für Gesundheit und Umwelt (GmbH), Germany. AsiSI-linearized targeting vector (15 μg) was introduced by electroporation into CCE ESCs, and clones were selected in the presence of G418 (200 μg/ml). Drug-resistant colonies were screened by Southern blotting using the restriction enzyme and probe combinations shown in Fig. 9A to identify correctly targeted clones (see Fig. 9B). Correctly targeted *Prdm4^+/TA^* ESCs were used to generate germ line chimeras, and the resulting heterozygous animals were intercrossed to obtain homozygous mice for tissue analysis. The *Prdm4^TA^* allele was excised and converted into the null *Prdm4^LacZ^* allele by crossing heterozygous males with *Sox2.Cre* female carriers (39). The following primers and cycling conditions were used for PCR genotyping: for the *Prdm4^TA^* allele, common primer, GCC ACA GCC ATG ACT ACC TT; wild-type primer, GGA GCT TGT AGG TGG GCT AA; mutant primer, AAA GCA ATA GCA TCA CAA ATT TCA; 58°C annealing temperature, 40 cycles; for the *Prdm4^LacZ^* allele, common primer, AAC TGC ATC AGT TTA TCC CCT A; wild-type primer, ACA TTT CTG GGG GCA GTT TT; mutant primer, AAA GCA ATA GCA TCA CAA ATT TCA. All animal experiments were performed in accordance with Home Office regulations.

### Generation of stably transfected ESCs expressing GFP-epitope-tagged full-length and ΔZF Prdm4.

The full-length *Prdm4* coding sequence was PCR amplified from FANTOM3 clone 4022401E08 using primers GATAGAATTCACCATGAATGACATGAACTTGAGC and TATCCCCGGGTTTATGTGCGGAGAGAGACTC to introduce EcoRI and SmaI restriction sites. Alternatively, to generate the pCAGGS-Prdm4ΔZF-EGFP (enhanced green fluorescent protein) expression vector, the *Prdm4^ΔZF^* coding sequences were cloned from doubly targeted ESCs via reverse transcription-PCR (RT-PCR). The PCR products were cloned into complementary sites of pEGFP-N2 (Clontech), subsequently excised using XhoI and NotI, and inserted into a modified version of pCAGGS (40) containing an internal ribosome entry site (IRES) puromycin resistance cassette. The SalI-linearized vectors were electroporated into gelatin-adapted CCE or *Prdm4^ΔZF/ΔZF^* ESCs, and puromycin-resistant clones were screened for EGFP expression by flow cytometry and Western blot analysis.

### SELEX.

To generate the recombinant Prdm4 zinc finger domain, the coding sequence was PCR amplified from IMAGE clone (ID 8862431) using the primers Forward (GAT AGG ATC CCA TGG GCC AAG CCA CAG CAA GGA AAG G) and Reverse (GAT ACT CGA GTT ATT AGG AGC TGG GCT CTT TGC AGG TCT TCA G) and cloned into XhoI and BamHI sites of modified pET28a (Novagen). The bacterially expressed recombinant protein was induced with 0.1 mM isopropyl-β-d-thiogalactopyranoside (IPTG) and purified, and systematic evolution of ligands by exponential enrichment (SELEX) was performed as previously described (41).

### Cell fractionation and Western blotting.

Total cell lysates were prepared and Western blot analysis was performed as described previously (41) using a mouse anti-GFP monoclonal antibody, JL-8 (Clontech; catalog no. 632381; 1:1,000). Alternatively, isolation of nuclear and cytoplasmic fractions for Western blot analysis was performed as described previously (42).

### EMSA.

Electrophoretic mobility shift assays (EMSAs) were performed as described previously (41). Nuclear complexes were resolved in 0.8% agarose gels, or alternatively, 5% acrylamide gels were used for analysis of His-tagged purified Prdm4. Oligonucleotides used for EMSA probes were as follows: for wild-type *Bahcc1*, GGC CTG GGT CGG CCC GCG GGG ATC CTG GAA ACC GTC CCC GGT TTA TCT CCT T and GGA AGG AGA TAA ACC GGG GAC GGT TTC CAG GAT CCC CGC GGG CCG ACC CAG G; for ΔGAAAC *Bahcc1*, GGC CTG GGT CGG CCC GCG GGG ATC CTG TCC CAC GTC CCC GGT TTA TCT CCT T and GGA AGG AGA TAA ACC GGG GAC GTG GGA CAG GAT CCC CGC GGG CCG ACC CAG G; for ΔCT *Bahcc1*, GGC CTG GGT CGG CCC GCG GGG ATC AGG GAA ACC GTC CCC GGT TTA TCT CCT T and GGA AGG AGA TAA ACC GGG GAC GGT TTC CCT GAT CCC CGC GGG CCG ACC CAG G; for ΔGGGG *Bahcc1*, GGC CTG GGT CGG CCC GCT TTT ATC CTG GAA ACC GTC CCC GGT TTA TCT CCT T and GGA AGG AGA TAA ACC GGG GAC GGT TTC CAG GAT AAA AGC GGG CCG ACC CAG G; for ΔGGGG/ΔCT *Bahcc1*, GGC CTG GGT CGG CCC GCT TTT ATC AGG GAA ACC GTC CCC GGT TTA TCT CCT T and GGA AGG AGA TAA ACC GGG GAC GGT TTC CCT GAT AAA AGC GGG CCG ACC CAG G. Antibody supershifting was performed by the addition of 0.1 μg of anti-Prdm4 rabbit polyclonal antibody (Sigma-Aldrich; HPA024322) or 1 μg anti-GFP mouse monoclonal antibody 3E6 (Invitrogen; A11120) to the binding reaction mixtures.

### Chromatin immunoprecipitation and deep sequencing (ChIP-seq).

Stably transfected ESC clones expressing full-length Prdm4-EGFP (FL-Prdm4-EGFP) (2 × 10^7^ to 3 × 10^7^ cells for each sample) were subjected to ChIP using either 6 μg of anti-GFP monoclonal antibody (Invitrogen; A11120, clone 3E6, IgG2a) or control mouse IgG (Santa Cruz; sc-2025, mouse IgG) as described previously (43). The resulting DNA samples were multiplexed and sequenced using two lanes on an Illumina HiSeq 2000 sequencer.

### Whole-genome ChIP-seq analysis.

Sequence reads were mapped to the mm9 mouse genome release with Stampy using default parameters (44). Peak calling was performed using MACS2 (45, 46), using default parameters to call areas of enrichment in the anti-GFP ChIP over the nonspecific whole-mouse IgG control ChIP. Overlapping peaks with a score of ≥10 in all four replicates were identified, and the core region of overlap was used for further analyses. The genomic distribution of ChIP-seq peaks compared to gene annotations was determined using CEAS (47). Genes of Ensembl release 67 with proximal Prdm4 binding were identified using custom Perl scripts. *De novo* motif finding within ChIP-seq peaks was performed using MEME (48). ChIP-seq peak coordinates were compared to Ensembl Regulatory Features of release 67 and ERV1:RLTR23 regions using custom Perl scripts. Functional annotation of Prdm4 ChIP-seq peaks was performed using GREAT version 2.0.2 using the basal plus extension rule, annotating genes within 5 kb of transcription start sites initially and within 1 Mb where no proximal gene exists (49). Terms with a binomial *P* value of ≤1 × 10^−5^ were considered significant.

For comparison, Smad2 ChIP-seq peak coordinates were downloaded from NCBI GEO accession numbers GSM578474 and GSM578475, and Klf5 ChIP-seq peak coordinates were acquired from the online version of the work of Parisi et al. (50). Genes with proximal Smad2 and Klf5 binding were identified as outlined above. The association between proximal binding and differentially expressed genes was calculated by chi-square test.

### Microarray experiments.

ESCs were washed and directly lysed in TRIzol (Invitrogen) on culture plates. RNA was extracted and Turbo DNase (Invitrogen) treated according to the manufacturers' instructions. The RNA was then cleaned using an RNeasy minikit (Qiagen) and hybridized to Illumina Mouse WG-6 v2 Expression BeadChips as described previously (4). Four biological replicates were performed for *Prdm4^ΔZF/ΔZF^* cells, and six biological replicates each were performed for wild-type and stably transfected ESCs expressing full-length Prdm4-EGFP.

### Microarray data analysis.

Differential expression was determined following rank-invariant normalization by using the Illumina custom error model option with Benjamini and Hochberg false discovery rate. Probes with an Illumina DiffScore of >30, equivalent to a *P* value of <1 × 10^−3^, were considered significant. Probes corresponding to genes in Ensembl release 67 were then compared to Prdm4 binding regions identified by ChIP-seq. Functional annotation analysis was performed using DAVID Informatics Resources 6.7 (51, 52). Enriched gene ontology biological process terms with Benjamini-Hochberg corrected *P* values of ≤2 × 10^−2^ were considered significant.

### qPCR.

Quantitative PCR (qPCR) was performed as previously described (42) with the exception that expression was normalized to *Actb* rather than *Hprt*. Primer sequences used are shown in Data set S1 in the supplemental material.

### Alkaline phosphatase assay.

Cells were plated at 200 cells per well in 6-well plates in the presence of LIF. Cells were then subsequently cultured in the presence or absence of LIF for 4 to 6 days and then stained with the alkaline phosphatase detection kit (Millipore) per the manufacturer's instructions.

### Immunofluorescence microscopy of embryonic stem cells.

ESCs were plated on gelatin-coated coverslips and cultured for up to 3 days. Cells were fixed with 4% paraformaldehyde and permeabilized with 0.2% Triton X-100–phosphate-buffered saline (PBS) before blocking with 10% donkey serum with 1% bovine serum albumin (BSA) and 0.05% Tween 20 in PBS. Primary antibodies used include goat anti-Oct4 (Santa Cruz; sc-8628) and rabbit anti-Nanog (Abcam; ab80892), followed by the appropriate conjugated secondary antibodies: anti-goat Alexa Fluor 488-conjugated antibodies and anti-rabbit Alexa Fluor 555-conjugated antibodies (Molecular Probes/Invitrogen). Coverslips were mounted with Vectashield mounting agent containing 4′,6-diamidino-2-phenylindole (DAPI; Vector Laboratories; H-1200). Fluorescent images were captured with a Leica epifluorescence microscope.

### Microarray data accession numbers.

The microarray and ChIP-seq data have been deposited in NCBI GEO with the accession numbers GSE46308 and GSE48372, respectively.

## RESULTS

### Prdm4 is widely expressed throughout the developing embryo and adult tissues.

**Fig 1:**
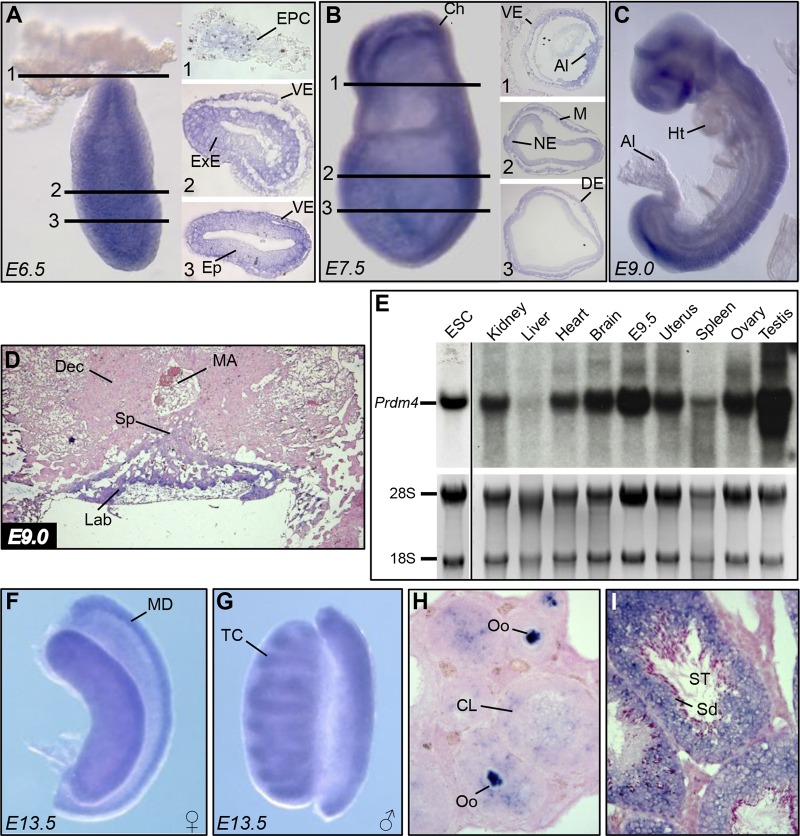
*Prdm4* RNA expression analysis during embryonic and postnatal development. (A) *Prdm4* whole-mount *in situ* hybridization at E6.5 shows widespread expression in all tissues, with the exception of the EPC. (B) At E7.5, *Prdm4* expression is apparent throughout the embryonic and extraembryonic tissues. (C) At E9.0, *Prdm4* is broadly expressed. (D) ISH of a midsagittal section through the forming placenta shows *Prdm4* expression confined to the developing labyrinth. (E) Northern blot assay on ESCs, 4-week-old mouse tissues, and E9.5 embryo using a radiolabeled probe spanning the entire *Prdm4* coding sequence. 28S and 18S bands are shown as loading controls. (F and G) Whole-mount ISH analysis of E13.5 female and male gonads shows that *Prdm4* is most prominently expressed in the Müllerian duct and forming testis cords, respectively. (H) In the adult ovary, high levels of expression are present in all stages of oocyte maturation and in the corpus luteum. (I) *Prdm4* transcripts are abundantly expressed in developing spermatozoa and supporting cells of the seminiferous tubules. Abbreviations: EPC, ectoplacental cone; VE, visceral endoderm; ExE, extraembryonic ectoderm; Ep, epiblast; Ch, chorion; Al, allantois; NE, neuroectoderm; M, mesoderm; DE, definitive endoderm; Ht, heart; Sp, spongiotrophoblast layer; MA, maternal artery; Dec, decidua; Lab, labyrinth layer; ESC, embryonic stem cells; MD, Müllerian duct; TC, testis cords; Oo, oocytes; CL, corpus luteum; ST, seminiferous tubules; Sd, spermatids.

Unlike Prdm1 and Prdm14 transcripts selectively expressed in germ cells at early postimplantation stages, single-cell profiling experiments revealed that Prdm4 transcripts were present at roughly equivalent levels in both the somatic and germ cell lineages (53). Similarly here, whole-mount *in situ* hybridization experiments demonstrate ubiquitous *Prdm4* expression throughout the embryo at E6.5 (Fig. 1A). A striking exception that lacks expression is the ectoplacental cone (EPC), a derivative of the extraembryonic ectoderm. At E7.5 (Fig. 1B) and slightly later at E9.0 (Fig. 1C), we similarly observe uniformly strong expression throughout the embryo proper. In the developing placenta, transcripts are readily detectable in the labyrinth layer, derived from the chorion and allantois, but absent in the spongiotrophoblast layer derived from the EPC (Fig. 1D).

Northern blot analysis demonstrates that Prdm4 mRNA is strongly expressed in ESCs and E9.5 embryos (Fig. 1E), and similar to findings in the developing embryo, we also detect expression in all adult tissues tested except the liver (Fig. 1E). Reproductive tissues, including the testes, ovaries, and uterus, show particularly high levels of expression. Next, we performed whole-mount and section *in situ* hybridization on pre- and postnatal gonads. Both the somatic and germ cell components of the developing gonads of both sexes express Prdm4 transcripts (Fig. 1F and G and data not shown). We observe the strongest signal in the Müllerian duct mesenchyme and in the condensing testis cords. In adult ovaries, robust expression of Prdm4 marks oocytes at all stages of their maturation as well as the somatic cells of the corpora lutea (Fig. 1H). In the testis, Prdm4 expression in the seminiferous tubules marks all stages of the developing spermatozoa as well as the Sertoli cells (Fig. 1I).

### Prdm4 binds a tripartite recognition sequence in close proximity to TSSs.

**Fig 2:**
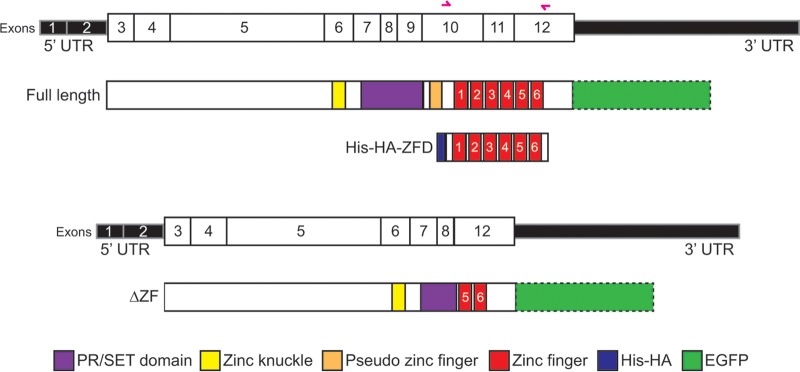
Illustration of the *Prdm4* cDNA-protein structural domain relationships. The *Prdm4* transcript with numbered exons (top) is aligned with full-length Prdm4 protein and the recombinant and mutant proteins represented in this study. The protein that results from deletion of exons 9 to 11 (ΔZF) is shown, as well as the recombinant His-HA-tagged zinc finger domain protein used for SELEX and EMSA. Primer sites for cloning the zinc finger domain are shown as pink arrows. C-terminal EGFP (in outline) denotes tagged protein expressed in stably transfected ESCs, as used for ChIP-seq, Western blot analysis, and imaging. UTR, untranslated region.

**Fig 3:**
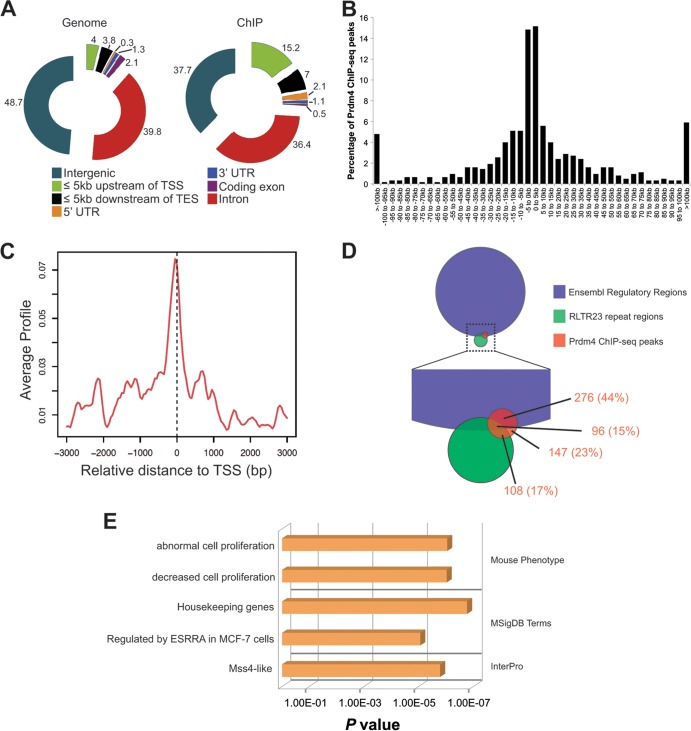
Prdm4 binds to known regulatory elements in close proximity to transcription start sites. (A) Distribution of Prdm4 binding events identified by ChIP-seq relative to gene annotations (right) compared to all genomic regions (left) in each category as indicated by the key. Charts are annotated with the percentage of the genome and the percentage of ChIP-seq-identified regions in each genomic category. Abbreviations: TSS, transcription start site; TES, transcription end site. (B) The distance of each Prdm4 ChIP-seq peak from the nearest TSS binned at 5-kb intervals. (C) The average ChIP-seq enrichment signal around TSSs of genes indicating bias for Prdm4 binding at TSSs. (D) Venn diagram indicating the percentage of Prdm4 ChIP-seq peaks within annotated Ensembl regulatory features and ERV1:RLTR23 repeat sequences. (E) Significant terms associated with Prdm4 binding identified by GREAT.

We performed ChIP-seq experiments using stably transfected ESCs strongly expressing full-length Prdm4-EGFP from the chicken beta-actin promoter in combination with a ChIP-quality anti-GFP monoclonal antibody (30) (Fig. 2). Duplicate experiments performed on two independent Prdm4-EGFP-expressing ESC clones identified a total of 627 Prdm4 binding regions (see Data set S2 in the supplemental material). Analysis of their genomic distribution relative to gene annotations (Fig. 3A), together with assessment of the absolute distances relative to the nearest transcription start site (TSS) (Fig. 3B), revealed a significant enrichment within 5 kb of transcription start sites in either direction (*P* = 8.3 × 10^−29^). Visualization of the average binding profile around TSSs demonstrates a particular bias to binding fractionally upstream of the TSS (Fig. 3C). Genes exhibiting proximal Prdm4 binding are shown in Data set S2 in the supplemental material.

Next, we compared our data to known *cis*-regulatory elements. In particular, we examined Ensembl Regulatory Features, comprised of regions known to have histone modifications associated with functionally active chromatin, DNase I hypersensitivity peaks, and transcription factor binding sites described in published ChIP-seq experiments (54). These results demonstrate that 59% (372/627) of our Prdm4 ChIP-seq peaks map within known regulatory regions (Fig. 3D; see also Data set S3 in the supplemental material). Interestingly, 33% (204/627) occur within ERV1:RLTR23 repeat regions (Fig. 3D). In some cases, mouse-specific families of transposable elements have been implicated in remodeling of the transcriptional circuitry in mouse ESCs, but the specific role of these ERV1:RLTR23 repeat regions has yet to be elucidated (55).

Finally, to gain further insights into these Prdm4 binding events, we performed functional annotation. GREAT analysis (Fig. 3E) revealed relatively few significant terms associated with Prdm4 binding. However, proximal Prdm4 binding was found to be enriched among genes governing cell proliferation, housekeeping genes, and Mss4-like genes. Structural studies suggest that Mss4 may act as a guanine nucleotide-free chaperone. However, the functional activities contributed by the Mss4-like domain remain unknown (56–58).

**Fig 4:**
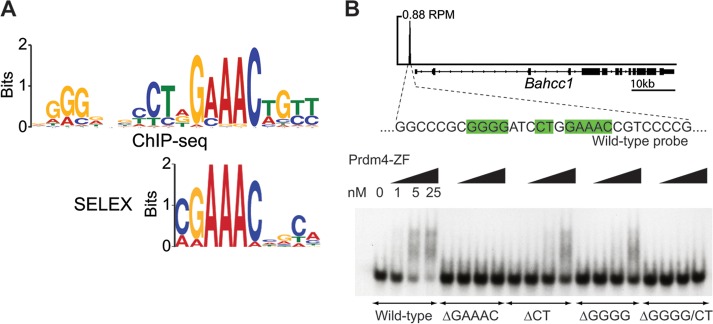
Prdm4 binds a tripartite recognition sequence. (A) Prdm4-EGFP ChIP-seq analysis reveals a consensus binding motif (top panel) similar to that identified by SELEX using cloned recombinant Prdm4-ZFD (bottom panel). (B) Prdm4 binds 1.1 kb upstream of the *Bahcc1* transcription start site. The corresponding region was used to generate an EMSA probe. Selective mutation of elements of the DNA binding motif (green) combined with EMSA reveals that the core motif identified by SELEX and ChIP-seq (GAAAC) is required for Prdm4-DNA interaction while the peripheral regions identified by ChIP-seq (GGGG and CT) augment DNA binding. RPM, peak height in reads per million.

In order to identify the sequence bound by Prdm4, we performed *de novo* motif finding. We identified three highly similar tripartite sequences of the form GGGn_(1–4)_CTnGAAAC within the Prdm4 binding regions (Fig. 4A; see also Fig. S1 in the supplemental material). Nearly all (602/627, 96%) ChIP-seq peaks contain at least one significant match to one of these motifs (see Fig. S1).

To strengthen these results, we generated recombinant Prdm4-ZFD and performed SELEX. A highly similar motif corresponding to the most conserved (GAAAC) region of sequence bound by Prdm4 was identified (Fig. 4A). We also performed EMSAs using the recombinant His-hemagglutinin (HA)-tagged Prdm4-ZFD. The control probe corresponding to the 50-bp central portion of the ChIP-seq peak upstream of *Bahcc1* contains a strong match for the Prdm4 15-mer binding motif (Fig. 4B). Variants with three discrete motif components mutated individually and in combination were tested to evaluate their relative contributions to Prdm4 binding (Fig. 4B). The GAAAC component of the binding motif appears to be essential for Prdm4-ZFD binding, whereas the CT and GGGG components, as predicted by their weaker conservation, modulate the strength of binding but are not individually required.

### Targeted deletion of the ZF domain results in a functional null allele.

**Fig 5:**
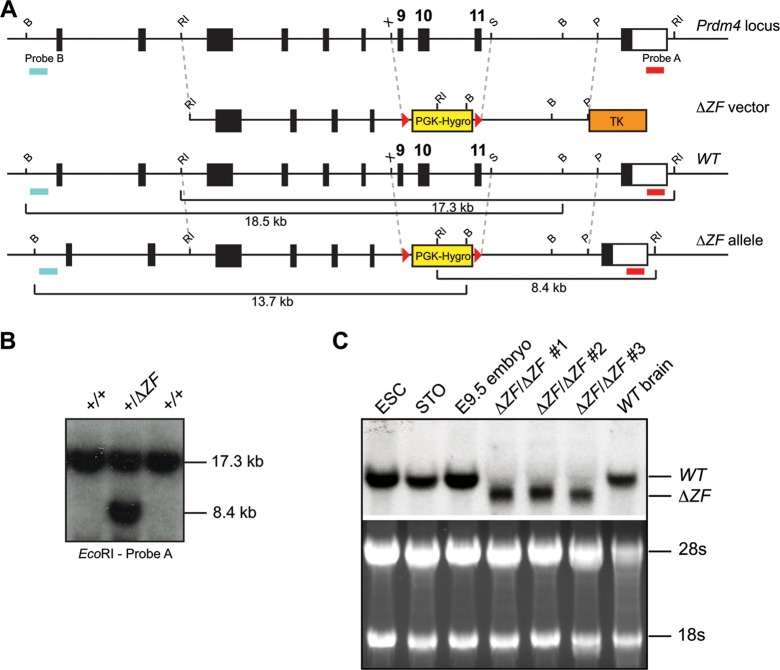
*Prdm4^ΔZF^* deletion allele targeting strategy. (A) Schematic representation of the wild-type (WT) locus, targeting vector, *Prdm4^ΔZF^* mutant allele, and Southern blot screening probes. B, BglII; P, PmeI; RI, EcoRI; S, SacI; X, XhoI. LoxP sites are represented by red arrowheads. (B) Southern blot analysis of representative drug-resistant colonies. The positions of diagnostic wild-type (17.3-kb) and targeted (8.4-kb) fragments are shown. (C) Northern blot analysis of *Prdm4* transcripts in wild-type and homozygous *Prdm4^ΔZF/ΔZF^* ESCs. Full-length (wild-type) and truncated Δ*ZF* RNA fragments are indicated.

EMSAs demonstrate that a recombinant His-HA-tagged Prdm4-ZFD on its own is sufficient to mediate DNA binding. Targeted deletion of the Prdm4 ZFD should therefore create a functional null allele. This strategy was successfully exploited to disrupt *Prdm1* functional activity *in vivo* (8, 59). We engineered a targeted deletion spanning a 3.25-kb fragment (chr10:85361568 to 85364815, NCBI37 assembly, mm9) encompassing exons 9, 10, and 11 (Fig. 2 and 5A) that encodes 67 C-terminal amino acids of the PR/SET domain, the pseudo Zn finger at position 548 to 569, and 4 proximal Zn fingers within the ZFD (amino acid positions 593 to 699) (Fig. 2) (Ensembl protein ENSMUSP00000041942). Correctly targeted cells were Cre excised to eliminate the *PGK-Hygro* selection cassette and subjected to retargeting to generate homozygous *Prdm4^ΔZF/ΔZF^* ESCs. These doubly targeted clones appear morphologically indistinguishable from wild-type ESCs.

**Fig 6:**
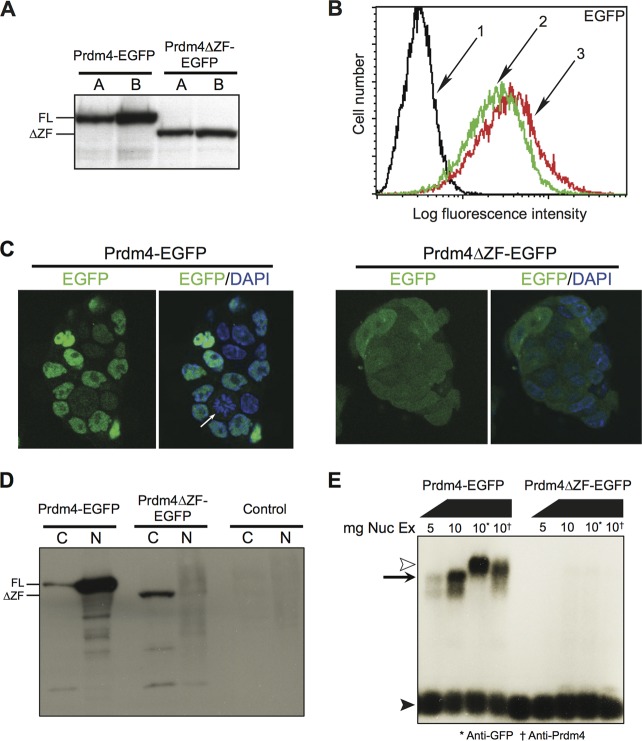
The truncated Prdm4ΔZF protein is predominantly cytoplasmic and fails to bind DNA. (A) Western blot analysis of whole-cell radioimmunoprecipitation assay buffer lysate. Shown are two clones each of Prdm4-EGFP-expressing wild-type ESCs and Prdm4ΔZF-EGFP-expressing *Prdm4^ΔZF/ΔZF^* ESCs. (B) Relative fluorescence of untransfected ESCs and Prdm4-EGFP-expressing and Prdm4ΔZF-EGFP-expressing ESCs. (C) Confocal imaging of stably transfected ESCs expressing full-length Prdm4-EGFP (clone A) or a truncated Prdm4ΔZF-EGFP variant (clone B). Full-length Prdm4-EGFP localizes to the nucleus and is only occasionally found in the cytoplasm of dividing cells lacking a nuclear membrane (white arrow). In contrast, Prdm4ΔZF-EGFP is predominantly cytoplasmic. (D) Nuclear and cytoplasmic extracts from Prdm4-EGFP clone A and Prdm4ΔZF-EGFP clone B were compared by Western blotting. (E) EMSA reveals that full-length Prdm4-EGFP strongly binds the *Bahcc1* promoter probe (left), whereas Prdm4ΔZF-EGFP cannot form complexes (right). Abbreviations and symbols: FL, full length; ΔZF, Prdm4ΔZF-EGFP; line 1, untransfected ESCs; line 2, Prdm4-EGFP clone A; line 3, Prdm4ΔZF-EGFP clone B; C, cytoplasmic fraction; N, nuclear fraction; black arrowhead, free probe; black arrow, probe:Prdm4-EGFP complex; white arrowhead, supershifted probe:Prdm4-EGFP complex.

Stably transfected *Prdm4^ΔZF/ΔZF^* ESCs exclusively expressing either FL-Prdm4-EGFP or the Prdm4ΔZF variant at equivalent levels (Fig. 2 and 6A and B) were examined by confocal microscopy (Fig. 6C). As expected, FL-Prdm4-EGFP-tagged protein predominantly localizes to the nucleus, whereas Prdm4ΔZF-EGFP remains cytoplasmic. A similar conclusion was reached via Western blot analysis of nuclear and cytoplasmic fractions (Fig. 6D). Moreover, EMSAs demonstrate that nuclear extracts from Prdm4ΔZF-EGFP-expressing cells lack DNA binding activity (Fig. 6E). A strong argument can therefore be made that the Prdm4ΔZF protein represents a functionally null variant.

### Prdm4 regulates *Nodal* and *Klf5* expression upstream of key pluripotency and differentiation pathways.

**Fig 7:**
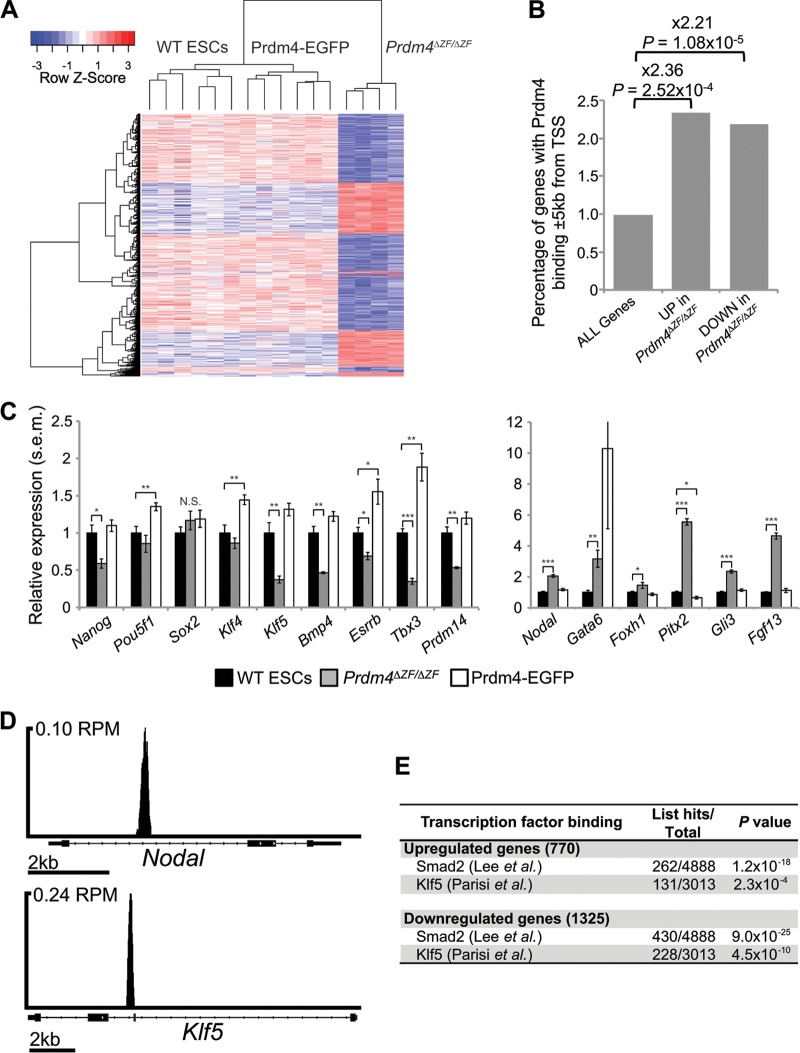
Prdm4 functional loss causes misregulated expression of pluripotency/differentiation genes, including genes with proximal Prdm4 binding. (A) Hierarchical clustering of microarray data reveals distinct transcriptional signatures between *Prdm4^ΔZF/ΔZF^* ESCs and wild-type and stably transfected cells expressing Prdm4-EGFP. Represented are the 2,095 genes differentially expressed between *Prdm4^ΔZF/ΔZF^* and wild-type ESCs (vertical) across the 16 independent samples (horizontal). The heat map represents standard score (the number of standard deviations removed from the average intensity per row). Samples with lower than the average intensity are blue; those with higher than the average intensity are red. (B) Genes either up- or downregulated in *Prdm4^ΔZF/ΔZF^* cells are significantly more likely to have Prdm4 binding within 5 kb of their TSS than are all genes. (C) qPCR shows that pluripotency markers are significantly downregulated in *Prdm4^ΔZF/ΔZF^* cells and upregulated in Prdm4-EGFP-expressing cells (left) whereas differentiation markers are upregulated in *Prdm4^ΔZF/ΔZF^* cells (right). *, *P* < 1 × 10^−2^; **, *P* < 1 × 10^−3^; ***, *P* < 1 × 10^−5^; N.S., not significant. (D) The TGF-β gene family member *Nodal* and pluripotency factor gene *Klf5* show proximal binding of Prdm4; TSSs are to the left. RPM, peak height in reads per million. (E) Up- and downregulated genes are significantly more likely to have Smad2 binding and Klf5 binding within 10 kb of the TSSs.

Next, we performed transcriptional profiling experiments comparing wild-type, stably transfected FL-Prdm4-EGFP-expressing ESCs and homozygous null *Prdm4^ΔZF/ΔZF^* ESCs. A large number of differentially expressed transcripts were identified in wild-type ESCs compared with *Prdm4^ΔZF/ΔZF^* mutant ESCs (770 upregulated; 1,325 downregulated; *P* ≤ 1 × 10^−3^) (Fig. 7A). In contrast, relatively few differences were observed in comparing wild-type with stably transfected Prdm4-EGFP-expressing cells (23 upregulated; 15 downregulated). Prdm4 loss of function therefore appears to have a substantial impact, whereas *Prdm4* overexpression has a less striking effect on gene expression patterns. Interestingly, both down- and upregulated genes are significantly more likely to have proximal Prdm4 binding than are all genes (Fig. 7B). These results strongly suggest that Prdm4 functions as both a transcriptional activator and a repressor.

Our functional annotation analysis of the differentially expressed genes revealed, among downregulated genes (GO:0045596, *P* = 1.4 × 10^−2^), significant enrichment for negative regulators of differentiation, including the key pluripotency genes *Pou5f1* (*Oct4*), *Nanog*, and *Bmp4* (Fig. 7C; see also Data set S4 in the supplemental material). Upregulated genes are significantly enriched for transcripts associated with embryonic development (GO:0043009, *P* = 1.7 × 10^−2^), including *Nodal*, *Gli3*, *Gata6*, *Pitx2*, and others (Fig. 7C; see also Data set S5 in the supplemental material). Interestingly, both *Klf5* and *Nodal* contain proximal Prdm4 binding sites (Fig. 7C and D).

*Nodal*, a member of the transforming growth factor β (TGF-β) superfamily of secreted growth factors, plays an essential role in anterior-posterior and left-right axis formation and mesodermal patterning in the early vertebrate embryo (60). Nodal/Activin signaling regulates ESC differentiation *in vitro* (61–63) and functions to maintain pluripotency through regulation of *Nanog* (64). Similarly, the zinc finger transcription factor *Klf5* is essential for ESC self-renewal (65) and functions redundantly in maintaining pluripotency by directly regulating expression of key target genes such as *Pou5f1* (*Oct4*), *Sox2*, and *Nanog* (66).

To test whether misregulated expression of *Klf5* and *Nodal* accounts for the transcriptional shift observed in *Prdm4^ΔZF/ΔZF^* ESCs, we interrogated published Klf5 and Smad2 (the downstream effector of Nodal signaling) ChIP data sets (50, 67). Genes with proximal Klf5 and Smad2 binding were compared to those differentially expressed in *Prdm4^ΔZF/ΔZF^* ESCs. This exercise reveals a highly significant association between occupancy by these transcription factors and genes misregulated in the absence of *Prdm4*. It is therefore tempting to speculate that Prdm4 may regulate *Klf5*- and *Nodal*-dependent pathways (Fig. 7E). Genes differentially expressed in *Prdm4^ΔZF/ΔZF^* cells with proximal binding of Klf5 or Smad2 are shown in Data sets S6 to S9 in the supplemental material.

Next, to test whether this altered transcriptional signature could potentially influence ESC differentiation dynamics, we examined *Oct4* and *Nanog* expression comparing wild-type, homozygous null *Prdm4^ΔZF/ΔZF^*, and stably transfected wild-type ESCs expressing either full-length Prdm4-EGFP or Prdm4ΔZF-EGFP. Consistent with the results above, cells maintained in the presence of LIF display robust expression of these pluripotency markers (see Fig. S2 in the supplemental material). Cells were subsequently cultured without LIF and then stained for alkaline phosphatase activity. As judged by morphological criteria, all the cells were induced to differentiate. However, a proportion of Prdm4-EGFP-overexpressing colonies retain ESC-like characteristics (see Fig. S2). Results of alkaline phosphatase staining similarly demonstrate that overexpression enhances ESC self-renewal. In contrast, Prdm4-deficient ESCs display increased ESC differentiation abilities. Collectively, these results support the idea that Prdm4 plays a regulatory role upstream of early developmental pathways.

### Prdm4 is nonessential for mouse development and fertility.

**Fig 8:**
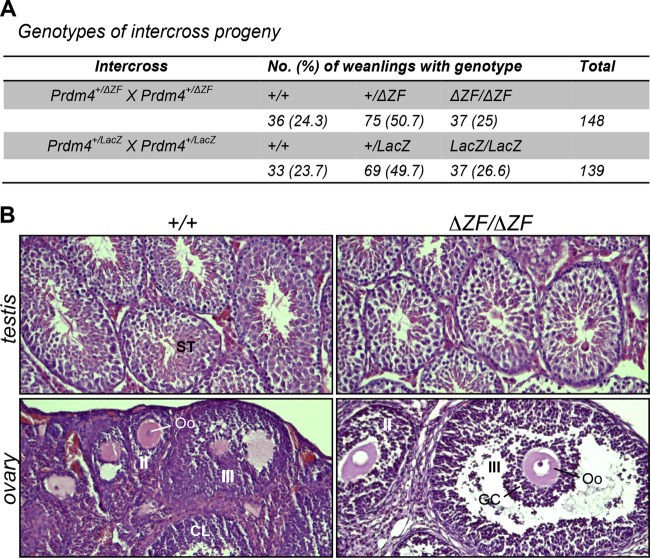
Prdm4 mutant mice are viable and fertile. (A) Intercross matings of heterozygous *Prdm4^+/^*Δ*^ZF^* and *Prdm4^+/LacZ^* animals generate Mendelian numbers of wild-type and heterozygous and homozygous mutant progeny. (B) Germ cell maturation and gonadal development are unperturbed in adult male and female Prdm4 mutant mice. Hematoxylin- and eosin-stained sections through the ovaries and testes of wild-type and mutant littermates fail to reveal defects in germ cell maturation. Abbreviations: ST, seminiferous tubules; CL, corpus luteum; II, secondary follicle; III, tertiary follicle; Oo, oocyte; GC, granulosa cells.

To further explore *Prdm4* functions *in vivo*, we generated mutant mice carrying the *Prdm4^ΔZF^* loss-of-function allele. *Prdm4^+/^*Δ*^ZF^* ESCs were used to generate germ line chimeras via blastocyst injection. However, subsequent heterozygous intercross matings resulted in homozygous mutant progeny at the expected Mendelian ratios (Fig. 8A). Homozygous mutants were indistinguishable from wild-type and heterozygous littermates and displayed no overt abnormalities.

**Fig 9:**
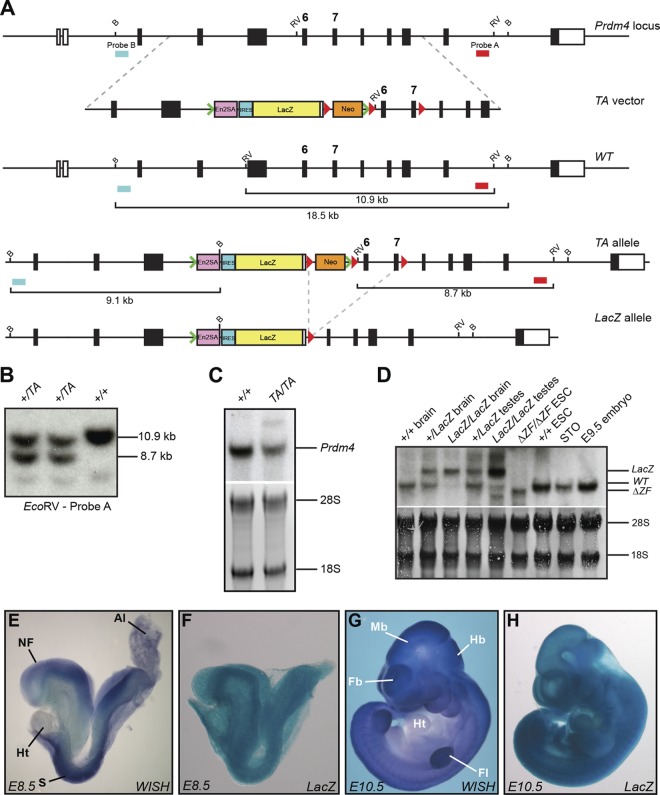
Generation of Prdm4EUCOMM targeted alleles. (A) Schematic representation of the wild-type locus, targeting vector, *Prdm4^TA^*, and *Prdm4^LacZ^* deletion allele. Southern blot screening probes are indicated. B, BglII; RV, EcoRV. LoxP sites are represented by red arrowheads, and FLP recombination target (FRT) sites are represented by green arrowheads. (B) Southern blot analysis of representative drug-resistant *Prdm4^TA^* colonies. The positions of diagnostic wild-type (10.9-kb) and targeted (8.7-kb) fragments are shown. (C) Northern blot analysis of wild-type and *Prdm4^TA/TA^* adult tissues shows the production of reduced levels of wild-type Prdm4 transcripts in homozygous tissue resulting from splicing around the SA-LacZ cassette. (D) Northern blot analysis of wild-type, heterozygous, and homozygous *Prdm4^LacZ^* mouse tissue shows that Cre excision of the Neo cassette and deletion of exons 6 and 7 result in the generation of a long Prdm4 transcript incorporating the *lacZ* cassette. The full-length and mutated RNA fragments are indicated. *Prdm4^ΔZF/ΔZF^* ESCs, wild-type ESCs, E9.5 embryo, and STO fibroblast RNAs were also included as controls. (E to H) LacZ and whole-mount ISH (WISH) staining of *Prdm4^+/TA^* and wild-type E8.5 and E10.5 embryos shows that LacZ staining faithfully recapitulates endogenous *Prdm4* staining. Abbreviations: NF, neural fold; Al, allantois; Ht, heart; S, somite; Fb, forebrain; Mb, midbrain; Hb, hindbrain; Fl, forelimb.

To confirm that Prdm4 is dispensable for mouse development, we also generated a second targeted allele using the EUCOMM/KOMP resource (68). Expression of the LacZ gene trap reporter allele faithfully recapitulates the endogenous *Prdm4* expression pattern in all tissues examined, including the developing embryo (Fig. 9E to H) as well as prenatal and postnatal gonads (data not shown). Northern blot analysis of tissue from wild-type and homozygous mice carrying the *Prdm4^TA^* allele shows significant splicing around the *lacZ* cassette and production of the wild-type *Prdm4* transcript (Fig. 9C). To obtain a null allele, heterozygotes carrying the *Prdm4^TA^* allele were crossed to *Sox2.Cre* partners to generate the *Prdm4^LacZ^* allele (Fig. 9A). This manipulation deletes exons 6 and 7, corresponding to amino acids 372 to 468 spanning the PR/SET domain, and results in a LacZ-Prdm4 fusion transcript completely lacking in-frame coding information downstream of the deletion (Fig. 9A). PCR genotyping confirmed the correct *Sox2.Cre*-mediated excision of the loxP site-flanked region. As described above, heterozygous intercross matings yielded homozygous mutant progeny at the expected Mendelian ratios (Fig. 8A). Northern blot analysis of total RNA from brain and testes of wild-type, heterozygous, and homozygous mutant mice confirmed the presence of the expected fusion transcript (Fig. 9D). Adult homozygous mutants failed to display any phenotypic disturbances. The present analysis of two independently generated *Prdm4* null alleles demonstrates that Prdm4 is nonessential for mouse development.

*Prdm4* is robustly expressed in both the somatic and germ cell components of the developing gonads (Fig. 1F to I), suggesting a potential role for Prdm4 in reproductive function. However, adult *Prdm4^LacZ/LacZ^* and *Prdm4^ΔZF/ΔZF^* homozygous mutants of both sexes (*n* = 4) test bred with wild-type partners proved fertile and gave rise to healthy litters. Consistent with this, both the ovaries and testes appear histologically normal and are indistinguishable from those of wild-type littermates (Fig. 8B). We found comparable numbers of developing follicles in the *Prdm4^ΔZF/ΔZF^* ovaries and no evidence for abnormal spermatogenesis in the mutant testes. These findings demonstrate that Prdm4 is also dispensable for germ cell development and fertility.

## DISCUSSION

The *Prdm* gene family first appeared in metazoans and experienced a massive expansion in vertebrates (2). In some cases, functions appear to be well conserved. For example, *Drosophila melanogaster hamlet* and closely related mammalian Prdms 8, 12, and 13 function downstream of the Notch-Hes pathway to control cell fate during neurogenesis (69, 70). On the other hand, striking species-specific differences have also been described. For example, *Blimp1/Prdm1* is essential for specification of the germ cell lineage in mice but not in fish (7, 9). In fish, *Blimp1/Prdm1* is required during specification of the slow twitch muscle cell lineage and a subset of sensory neurons (71–73), but in contrast, *Blimp1/Prdm1* has no known function in either muscle or neural crest lineages in mice (74).

At the amino acid level, Prdm4 is highly conserved across mammals (96% sequence identity between mouse and rat, 94% sequence identity between mouse and human). Previous studies have described *Prdm4* activities in the developing nervous system in rodents (32, 33, 36), but only limited information is available about its functional contributions elsewhere. Here, we document widespread *Prdm4* expression throughout the developing mouse embryo from postimplantation stages onwards, and in nearly all adult tissues except the liver. Strong expression was observed in the reproductive organs, namely, the testes and ovaries. Developing spermatozoa and maturing oocytes express abundant levels of *Prdm4* transcripts. Nonetheless, we found that *Prdm4* loss-of-function mutant mice develop normally, are healthy and fertile, and display no detectable phenotypic abnormalities.

*Prdm4* was initially cloned as a candidate tumor suppressor gene in human cancers (75) and characterized as a cytoplasmic effector molecule acting downstream of the p75 neurotrophin receptor in response to nerve growth factor signaling (32). The present experiments confirm that nuclear localization depends on the Prdm4 ZFD (36), and as expected, Prdm4 DNA binding activities are mediated by its zinc fingers. In the case of Blimp1/Prdm1, the zinc finger domain also serves as a binding interface for recruitment of epigenetic modifiers, namely, G9a and HDAC1/2 (21). An alternative Δexon7 isoform, having a drastically truncated zinc finger domain, lacks DNA binding activity and fails to bind to G9a or HDAC1/2 (41). Similarly here, the Prdm4ΔZF protein lacks the ability to tightly associate with chromatin inside the nucleus. As for the alternative Blimp1/Prdm1 Δexon7 isoform (41), the Prdm4ΔZF allele engineered here by homologous recombination in ES cells represents a loss-of-function mutation.

The present SELEX experiments demonstrate that the ZF domain is sufficient on its own to bind the Prdm4 tripartite consensus motif. Complementary ChIP-seq experiments revealed that 96% of target sites occupied by Prdm4 contain this consensus binding motif. Thus, Prdm4 DNA binding is predominantly sequence specific and independent of interactions with other transcription factors. Previous work suggested that Prdm4 functions as a transcriptional repressor through recruitment of HDACs (36). However, here integration of ChIP-seq and expression microarray data reveals that both upregulated and downregulated genes display Prdm4 binding sites. These results strongly suggest that Prdm4 mediates both activation and repression of target genes.

The *cyclin E* (CCNE1) gene was previously identified as a candidate Prdm4 transcriptional target (36). Small interfering RNA (siRNA) knockdowns resulted in increased *cyclin E* expression, and downregulated expression of a luciferase reporter construct containing the CCNE1 1.4-kb regulatory region was observed in transient-transfection assays (36). However, the present ChIP-seq experiments provided no evidence for proximal Prdm4 binding to *Ccne1* in ESCs. The 1.4-kb CCNE1 regulatory region (36, 76) lacks the Prdm4 consensus motif, and *Ccne1* expression was not found to be downregulated in stably transfected Prdm4-EGFP-expressing cells. Surprisingly, we observe that *Ccne1* is significantly downregulated in functional null *Prdm4^ΔZF/ΔZF^* cells (*P* = 8 × 10^−8^). The CCNE1 regulatory region does contain GAAAC—the minimal sequence motif identified in SELEX experiments. However, the larger tripartite motif present in 96% of ChIP-seq peaks and the broader motif elements identified in EMSAs taken together strongly suggest that GAAAC on its own is insufficient for Prdm4 binding. One possible scenario is that Prdm4 has the ability to recognize this minimal sequence motif due to increased *Prdm4* expression levels and target availability in transiently transfected cells. However, we find the opposing effects, namely, *Prdm4*-mediated repression of *cyclin E* expression versus decreased expression seen here in *Prdm4*-deficient ESCs, very perplexing. Collectively, these observations suggest that cell-type-specific chromatin context could have a dramatic impact on transcriptional output at the *Ccne1* locus.

Recent work suggests that *Prdm4* regulates cell cycle progression in neural stem cells (NSCs) (33). Thus, siRNA knockdown of *Prdm4* cultured embryonic cortical NSCs led to precocious differentiation. Here, in the absence of LIF *Prdm4^ΔZF/ΔZF^*-null ESCs display increased differentiation abilities. However, *Prdm4* loss-of-function mutant embryos develop normally, and adult homozygous mutants display normal body and organ size and are fertile. Moreover, expression microarray analysis of *Prdm4^ΔZF/ΔZF^* ESCs reveals misregulation of many key pluripotency genes. Genes with proximal Smad2 and Klf5 binding significantly overlap genes misregulated in *Prdm4^ΔZF/ΔZF^* cells. Both *Nodal* and *Klf5* contain proximal Prdm4 binding sites, strongly suggesting that Prdm4 functions upstream of *Nodal* and *Klf5* in the maintenance of pluripotency.

Dose-dependent Nodal/Smad2/3 signaling plays essential roles in the early mouse embryo (77, 78). The strength of Nodal/Smad2/3 signaling is tightly controlled by reciprocal feedback and feed-forward regulatory circuits between the embryo and extraembryonic tissues (79). The *cis*-regulatory enhancer elements directing dynamic patterns of *Nodal* expression have been extensively analyzed via complementary transgenic analysis and targeted deletion strategies (77, 78, 80). Interestingly, the Prdm4 binding site identified here lies near the intronic autoregulatory ASE (80). Recent studies demonstrate that continuous Nodal signaling actively recruits the histone demethylase Jmjd3 to the asymmetric *cis*-regulatory element to counteract repression by PRC2 (81). Prdm4 binding potentially functions collaboratively with Smad2/3 to maintain *Nodal* expression and antagonize PRC2-mediated repression.

*Klf5* is ubiquitously expressed in the preimplantation embryo, and in the trophectoderm lineage, *Klf5* is required to support implantation and expansion of the inner cell mass (ICM) (65). *Klf5*-deficient ESCs derived by sequential gene targeting precociously undergo differentiation, consistent with the idea that *Klf5* maintains pluripotency networks (65). The regulatory elements that drive *Klf5* expression in the early embryo remain unknown. The ChIP-seq experiments presented here demonstrate that Prdm4 occupancy overlaps with Esrrb, Ctcf, and Tcfcp2l1 binding sites (82), suggesting that this represents a key regulatory region driving *Klf5* expression in ESCs.

Recent experiments suggest that *Prdm14* functions to ensure pluripotency in cultured ESCs (24) and maintain so-termed naive pluripotency in the ICM (83). Here, we observe that *Prdm14* is significantly downregulated in functionally null *Prdm4^ΔZF/ΔZF^* ESCs (Fig. 7C) (*P* = 2 × 10^−13^). A recent genome-wide analysis of bivalent chromatin marks associated with expression of developmental genes poised for activation revealed that the epigenetic status of extraembryonic ectoderm and visceral endoderm tissues isolated from the early embryo only partially overlaps with that seen in the corresponding trophoblast stem cells (TS cells) and extraembryonic endoderm (XEN) cell lines (84). Similarly, *Prdm14* expression in the early embryo is strictly confined to the emerging primordial germ cells (PGCs), consistent with its essential role in governing their specification (10).

The present experiments demonstrate that *Prdm4* regulates transcriptional output in ESCs. However, *in vivo* in the context of the developing embryo and adult mouse, *Prdm4* is entirely dispensable. Additional work will be necessary to explore *Prdm4* functional contributions to tissue homeostasis, maintenance of hematopoietic stem cells, lymphocyte differentiation, and its possible roles in cancer. We are especially curious to learn more about *Prdm4* activities in the central nervous system. It will be important to evaluate whether, as in *Prdm8* mutant mice (85), neurogenesis in *Prdm4^ΔZF/ΔZF^* mice may be compromised, leading to subtle behavioral abnormalities. Currently, both the Prdm4-LacZ gene trap and *Prdm4^ΔZF/ΔZF^* functional null mutations are being maintained on a mixed C57BL/6J:129 genetic background. Extensive backcrossing will be required before proper behavioral and physiological phenotyping studies can be undertaken.

## Supplementary Material

Supplemental material
