# Supplementary material for: The PR/SET Domain Zinc Finger Protein Prdm4 Regulates Gene Expression in Embryonic Stem Cells but Plays a Nonessential Role in the Developing Mouse Embryo
Source: Mol Cell Biol. 2013 Oct;33(19):3936–50. doi: 10.1128/MCB.00498-13 (PMC3811882; doi:10.1128/MCB.00498-13)
Supplement: Supplemental material [file supp_33_19_3936__index.html]

Supplemental material 

# The PR/SET Domain Zinc Finger Protein Prdm4 Regulates Gene Expression in Embryonic Stem Cells but Plays a Nonessential Role in the Developing Mouse Embryo

## Supplemental material

**Files in this Data Supplement:**

- Supplemental file 1 -

  Fig. S1 (Sequence motifs enriched in Prdm4 ChIP-seq peaks) and S2 (Prdm4 effects on ESC differentiation and self-renewal)

  PDF, 3.1M
- Supplemental file 2 -

  Data sets S1 (qPCR primers), S2 (Genes with proximal Prdm4 binding), S3 (Ensembl regulatory features associated with Prdm4 ChIP-seq peaks), S4 and S5 (Genes involved in negative regulation of cell differentiation [S4] and chordate embryonic development [S5]), S6 and S7 (Genes upregulated [S6] and downregulated [S7] in Prdm4∆ZF/∆ZF with Klf5 binding), and S8 and S9 (Genes upregulated [S8] and downregulated [S9] in Prdm4∆ZF/∆ZF with Smad2 binding)

  XLS, 390K
