# Supplementary material for: The PR/SET Domain Zinc Finger Protein Prdm4 Regulates Gene Expression in Embryonic Stem Cells but Plays a Nonessential Role in the Developing Mouse Embryo
Source: Mol Cell Biol. 2013 Oct;33(19):3936–50. doi: 10.1128/MCB.00498-13 (PMC3811882; doi:10.1128/MCB.00498-13)
Supplement: Supplemental material [file MCB.00498-13_zmb999100141so1.pdf]

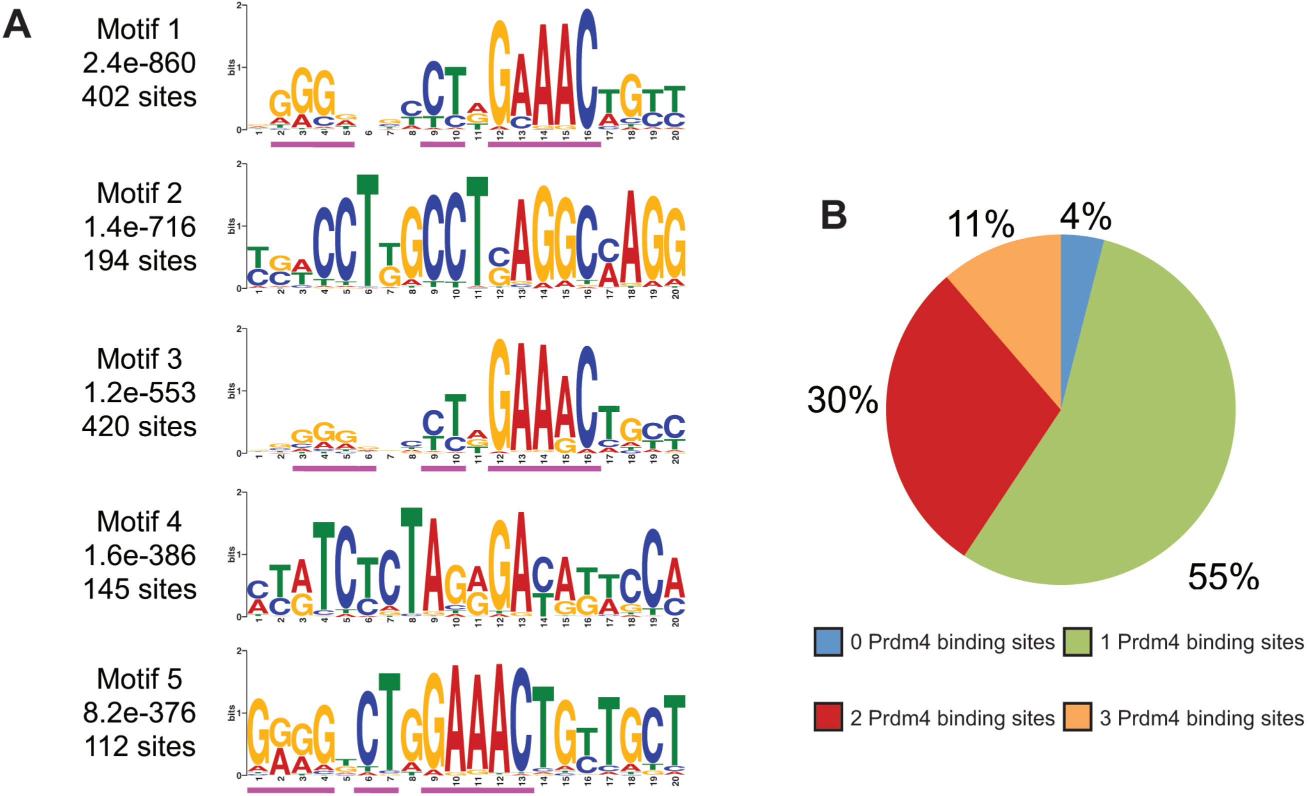
A
Motif 1
2.4e-860
402 sites
Motif 2
1.4e-716
194 sites
Motif 3
1.2e-553
420 sites
Motif 4
1.6e-386
145 sites
Motif 5
8.2e-376
112 sites
bits
B
11%
4%
30%
55%
0 Prdm4 binding sites
1 Prdm4 binding sites
2 Prdm4 binding sites
3 Prdm4 binding sites

FIG S1 Sequence motifs enriched in Prdm4 ChIP-seq peaks and their frequency. (A) Five 20mer sequences were identified within Prdm4 ChIP-seq peaks using MEME. *E* value and number of occurrences of each motif are shown. Motifs 1,3 and 5 are variants of the validated Prdm4-ZFD binding element. Motifs 2 and 4 are unidentified palindromes. The individual components of the Prdm4 tripartite motifs are underlined in pink. (B) Pie chart indicating number of significant matches ($P \leq 6.5 \times 10^{-4}$) to motif 1, 3 or 5 per ChIP-seq peak.

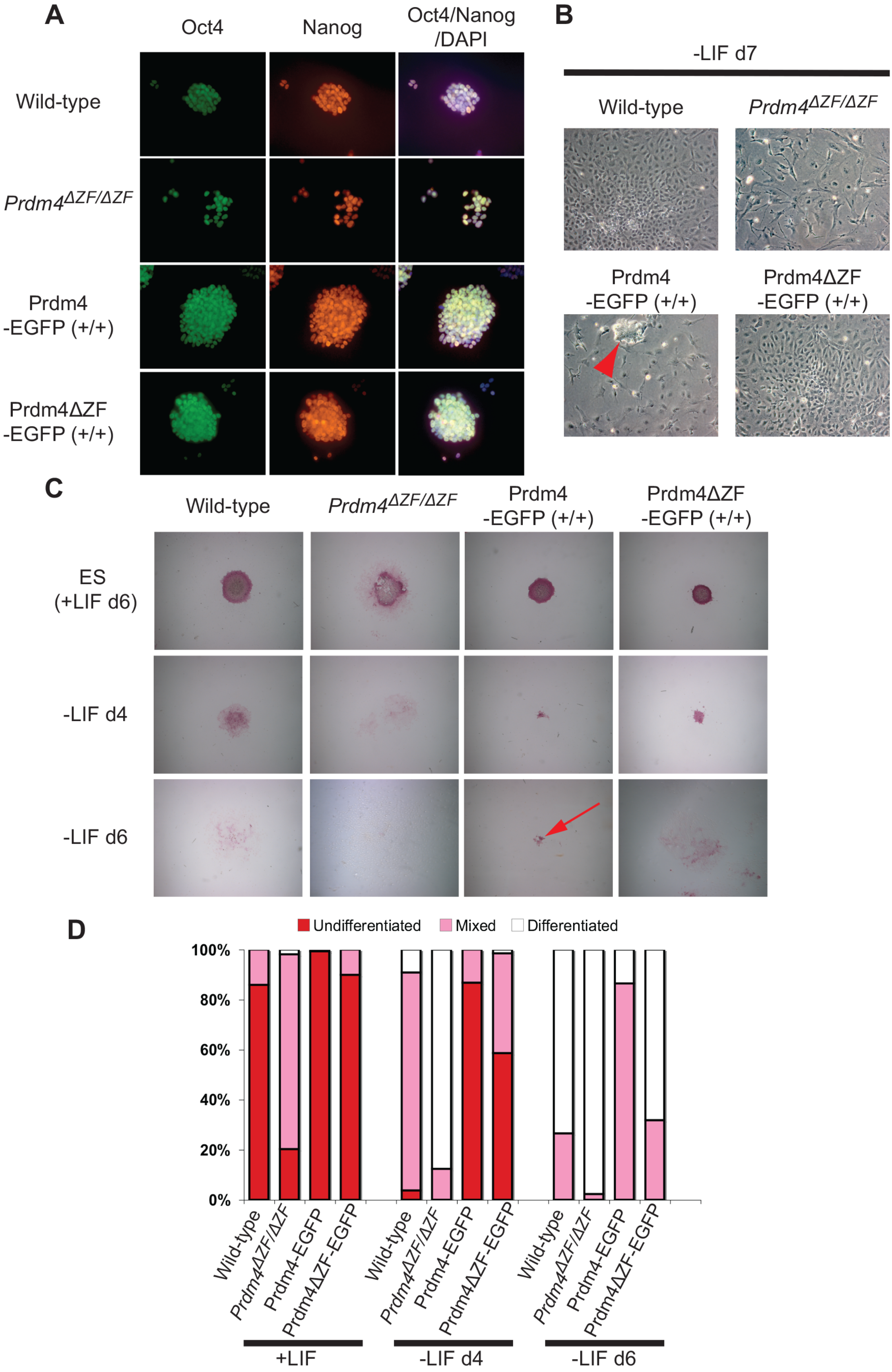

A
Oct4
Nanog
Oct4/Nanog /DAPI
Wild-type
Prdm4ΔZF/ΔZF
Prdm4 -EGFP (+/+)
Prdm4ΔZF -EGFP (+/+)
B
-LIF d7
Wild-type
Prdm4ΔZF/ΔZF
Prdm4 -EGFP (+/+)
Prdm4ΔZF -EGFP (+/+)
C
Wild-type
Prdm4ΔZF/ΔZF
Prdm4 -EGFP (+/+)
Prdm4ΔZF -EGFP (+/+)
ES (+LIF d6)
-LIF d4
-LIF d6
D
Undifferentiated
Mixed
Differentiated
100%
80%
60%
40%
20%
0%
Wild-type
Prdm4ΔZF/ΔZF
Prdm4-EGFP
Prdm4ΔZF-EGFP
+LIF
-LIF d4
-LIF d6

FIG S2 Loss of Prdm4 increases ESC differentiation abilities, while over-expression enhances self-renewal. (A) Expression of pluripotency markers Oct4 & Nanog in Prdm4-null (*Prdm4*$^{\Delta ZF/\Delta ZF}$) and wild-type ESCs, and wild-type cells over-expressing either EGFP tagged full-length Prdm4 (Prdm4-EGFP +/+) or EGFP-tagged Prdm4 lacking the zinc finger domain (Prdm4ΔZF-EGFP +/+). (B) ESCs were cultured without LIF (leukaemia inhibitory factor) for 7 days. All populations were induced to differentiate, but a proportion of Prdm4-EGFP over-expressing cells continue to display ESC-like morphology (red arrowhead), indicating that they may be more resistant to differentiation. (C & D) ESCs of indicated genotypes were plated at clonal density and grown in LIF-containing medium for 6 days (+LIF d6) or in the absence of LIF for 4 (-LIF d4) to 6 days (-LIF d6) and the resulting colonies were stained for alkaline phosphatase activity. Examples of clonal morphology are shown in (C). Colonies were classified into three categories as follows: Undifferentiated colonies (red bars) exclusively contained alkaline phosphatase positive cells. Differentiated colonies (white bars) gave no alkaline phosphatase staining. Mixed (pink bars) contained both alkaline phosphatase positive and unstained cells. (D) Bar graph summary of alkaline phosphatase staining results. Prdm4-null ESCs produced fewer 100% alkaline phosphatase positive colonies and rapidly differentiate upon LIF withdrawal. In contrast alkaline phosphatase positive cells continued to be observed in Prdm4-EGFP over-expressing cell cultures even upon LIF withdrawal for 6 days.
